# S-Adenosylmethionine and Superoxide Dismutase 1 Synergistically Counteract Alzheimer’s Disease Features Progression in TgCRND8 Mice

**DOI:** 10.3390/antiox6040076

**Published:** 2017-09-30

**Authors:** Rosaria A. Cavallaro, Vincenzina Nicolia, Maria Teresa Fiorenza, Sigfrido Scarpa, Andrea Fuso

**Affiliations:** 1 Department of Surgery “P. Valdoni”, Sapienza University of Rome, Via A. Scarpa 14, 00161 Rome, Italy; rosaria.cavallaro@uniroma1.it (R.A.C.); vincenzina.nicolia@hotmail.it (V.N.); sigfrido.scarpa@uniroma1.it (S.S.); 2 Division of Neuroscience, Department of Psychology, Sapienza University of Rome, Via dei Marsi 78, 00183 Rome, Italy; mariateresa.fiorenza@uniroma1.it; 3 IRCCS Santa Lucia Foundation, Via del Fosso di Fiorano 64-65, 00143 Rome, Italy

**Keywords:** Alzheimer’s disease, oxidative stress, S-adenosylmethionine, superoxide dismutase, one-carbon metabolism

## Abstract

Recent evidence emphasizes the role of dysregulated one-carbon metabolism in Alzheimer’s Disease (AD). Exploiting a nutritional B-vitamin deficiency paradigm, we have previously shown that PSEN1 and BACE1 activity is modulated by one-carbon metabolism, leading to increased amyloid production. We have also demonstrated that S-adenosylmethionine (SAM) supplementation contrasted the AD-like features, induced by B-vitamin deficiency. In the present study, we expanded these observations by investigating the effects of SAM and SOD (Superoxide dismutase) association. TgCRND8 AD mice were fed either with a control or B-vitamin deficient diet, with or without oral supplementation of SAM + SOD. We measured oxidative stress by lipid peroxidation assay, PSEN1 and BACE1 expression by Real-Time Polymerase Chain Reaction (PCR), amyloid deposition by ELISA assays and immunohistochemistry. We found that SAM + SOD supplementation prevents the exacerbation of AD-like features induced by B vitamin deficiency, showing synergistic effects compared to either SAM or SOD alone. SAM + SOD supplementation also contrasts the amyloid deposition typically observed in TgCRND8 mice. Although the mechanisms underlying the beneficial effect of exogenous SOD remain to be elucidated, our findings identify that the combination of SAM + SOD could be carefully considered as co-adjuvant of current AD therapies.

## 1. Introduction

Alzheimer’s disease (AD) is the most common age-associated progressive neurodegenerative disorder accounting for approximately 30 million people suffering worldwide; due to demographic changes and the longer life expectancy, this number is foreseen to increase up to 115 million cases in 2050 [1,2,3,4].

Most of the AD cases are sporadic, characterized by late onset (LOAD, Late Onset AD) and slow progression. AD shows typical brain neuropathological features including extracellular deposits of β-amyloid (Aβ) protein (senile plaques) and intracellular fibrillar deposits of hyper-phosphorylated tau protein (neurofibrillary tangles, NFT), leading to neuronal death and brain atrophy [5,6].

LOAD was recently described as a multifactorial disease likely resulting from the interaction between genetic, epigenetic and environmental factors able to shift physiological aging toward pathological processes [5,7,8]. These inducing factors include diet, nutritional lifestyle, hyperhomocysteinemia and oxidative stress, which is known to play a key role in the pathogenesis of LOAD, since it is thought to be one of the earliest events in the disease [9,10,11,12,13].

Indeed, consistent evidence supports the involvement of oxidative stress as a contributing factor in physiological aging and in the progression of multiple neurodegenerative pathologies [11,13] as well as in the etiopathogenesis of AD [2,14,15]. Markers of oxidative damage including extensive lipid peroxidation, high levels of oxidized proteins, oxidative modifications in RNA and nuclear and mitochondrial DNA, aldehydes, were found in the blood, in cerebrospinal fluid (CSF) and post mortem brains of neurological patients with preclinical or early stage AD [3,16,17,18,19,20].

Furthermore, in vitro and in vivo studies underline that oxidative stress affects Aβ production and oligomerization, which in turn generates free radicals, which in turn leads to a vicious cycle exacerbating the neurodegeneration process [2,21,22,23].

The brain accounts for 20% of all the oxygen and 25% of all glucose consumed by the body, despite it being only about 2% of body weight [24]. These characteristics make the human brain the organ most prone to aging and vulnerable to oxidative damage. This is due to the high concentration of easily oxidable polyunsaturated fatty acids (PUFAs) and the high concentration of iron and metals, which mediates the production of reactive oxygen species (ROS) [14,25], together with the relative deficiency of the antioxidant system. In this mechanism the blood brain barrier, since it needs to be bypassed by circulating antioxidants, plays a very important role.

Under normal physiologic conditions, ROS and reactive nitrogen species (RNS) are scavenged by a very effective cellular defense mechanism, using non-enzymatic and enzymatic antioxidants, including Glutathione peroxidase (GPx), catalase and superoxide dismutase (SOD). SOD catalyzes the conversion of O_2_^−^ to O_2_ and H_2_O_2_, more stable and with a lower oxidizing power, which is further transformed in H_2_O by catalase. There are two principal types of SOD: copper/zinc SOD (CuZn-SOD) that includes SOD1, localized in the cytosol and the extracellular SOD3, and manganese SOD (Mn-SOD) SOD2, localized in the mitochondrial matrix.

Frequently, LOAD is landmarked by a decrease in the activity and/or the levels of antioxidant enzymes including Glutathione reductase, Glutathione peroxidase and Glutathione-S-transferase and SOD [23,26,27,28].

SOD enzymes play an essential role for neural survival and protection against oxidative damage. It was shown that *Sod1* deficiency in the Tg2576 AD mouse model accelerated AD-like features, Aβ aggregation and tau hyperphosphorylation [28]; additional studies in the same mouse model showed that overexpression of SOD2 was able to prevent memory deficits and amyloid plaque deposition [29].

**Figure 1:**
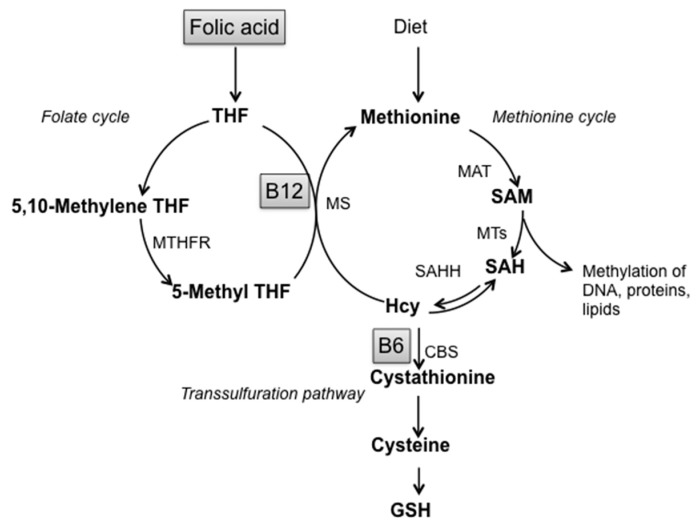
Schematic representation of one-carbon metabolism. Abbreviations: SAM: S-adenosylmethionine; SAH: S-adenosylhomocysteine; Hcy: Homocysteine; GSH: Glutathione; THF: Tetrahydrofolate; B12: Vitamin B12; B6: Vitamin B6; MTHFR: Methylenetetrahydrofolate reductase; MS: Methionine synthase; MAT: Methionine adenosyltransferase; MTs: Methyltransferases; SAHH: S-adenosylhomocysteine hydrolase; CBS: Cystathionine-β-synthase.

Non-enzymatic factors acting as scavengers are glutathione (GSH), nicotinamide adenine dinucleotide phosphate reduced form (NADPH), ascorbic acid, tocopherols and carotenoid derivatives. [11,27]. Alterations of GSH homeostasis, the most prevalent antioxidant thiol in brain, underline the important involvement of sulfur amino acids metabolism in the cellular response against oxidative stress. GSH participates in the metabolic pathway of Homocysteine (Hcy), defined as “one-carbon metabolism” that involves three interrelated biochemical pathways: the folate cycle, the transsulfuration pathway and the methionine cycle, leading to the production of S-adenosylmethionine, the main biological methyl donor in transmethylation reactions (Figure 1) [30,31].

Thus, this pathway represents the link between sulfur amino acid metabolism and the provision of methyl groups: indeed, alterations in the transsulfuration pathway cause loss of cellular redox homeostasis and reduced GSH production and are strictly connected with methylation cycle impairment through Hcy and S-adenosylhomocysteine (SAH) accumulation. Moreover, many studies evidenced that one-carbon metabolism alterations, low methylation status and oxidative stress are all linked to LOAD [6,10,32,33].

We extensively studied one-carbon metabolism alterations induced by B vitamin (folate, B12, and B6) deficiency in TgCRND8 mice overexpressing mutated human Amyloid Precursor Protein (APP) transgene and showing early Aβ plaque deposition [34]. We demonstrated that SAM counteracts the observed exacerbation of AD-like features induced by B vitamin deficiency, increasing both DNA methylation and antioxidant response. Specifically, SAM supplementation contrasts PSEN1 and BACE1 overexpression induced by B-vitamin deprivation, reduces β- and γ-secretase activities, Aβ production and plaques burden, restores PP2A activity and normal levels of Tau phosphorylation. and improves spatial memory in TgCRND8 mice [35,36,37,38].

We also showed that SAM prevents oxidative stress associated to one-carbon metabolism alteration, decreasing lipid peroxidation (LPO) levels by modulating GSH metabolism and SOD activity [39], corroborating the idea that one-carbon metabolism represents the link between DNA methylation and oxidative stress. Since the alteration of thiols content is influenced by the cellular response to oxidative stress injury, we also evaluated the effect of SAM and SOD supplementation either alone or in combination, on plasma thiols level, observing that their level significantly decreased when the mouse diet was supplemented with SAM + SOD and SOD alone [40].

Although a role for antioxidant agents in the prevention and/or treatment of AD has been hypothesized and supported by promising results in animal models [2,41,42], their beneficial effect on our species, as determined in clinical trials, appeared less convincing because of controversial results [4,43,44,45].

We therefore decided to investigate the possibility that SAM and SOD synergistically counteract the exacerbation of AD-like features enhanced by B-vitamin deficiency, by ameliorating both DNA methylation and oxidative balance.

## 2. Materials and Methods

### 2.1. Mice and Diets

TgCRND8 mice carrying the double mutant gene for the amyloid precursor protein APP695 (KM670/671NL_V717F) were obtained by D. Westaway (University of Toronto) and maintained by mating Tg male with non-Tg (TgCRND8^+/−^) 129-Sv female mice (Charles River Laboratories) as previously described [38]. At approximately 3 weeks of age, mice were genotyped and an equal number of Tg and 129Sv wild type (WT) littermate mice were randomly assigned to either control diet, to B vitamin-deficient diet (B deficient) and to B deficient diet supplemented with SAM (S-adenosylmethionine disulfate *p*-Toluensulfonate), SOD or SAM and SOD in combination (provided by Gnosis Spa, Desio MB, Italy), as previously described [40]; SAM and SOD were premixed to the diet pellets (Mucedola S.R.L., Settimo Milanese MI, Italy). Gastroresistant SOD powder composition is detailed in Supplementary Table S1. Diets lacking SOD (Control diet, B deficient diet and SAM-supplemented diet) were mixed with the same gastroresistant formulation described in Supplementary Table S1 except for SOD presence. All the experiments were performed on mice fed with the SAM and SOD supplemented diet, except for the uptake assay, as described. An equal number of male and female mice were assigned to each group; no gender differences were ever observed in any of the performed assay. SAM was added at a concentration of 0.1 g/kg to obtain a final dosage of 400 μg/day and SOD, in gastroresistant formulation, was added at 1250 U/kg to obtain a final dosage of 5 U per day. When used in combination SAM and SOD final dosages were 200 µg/day and 2.5 U/day, respectively. 1% sulphatiazole was added to each diet in order to prevent folate production by gut bacteria. Each experimental group included 8 mice (equal number of females and males) and the treatment with control diet or the various diet formulations described above was started at weaning and lasted 2 months. Brains and blood samples were collected and processed as previously described [38].

To obtain brains for immunohistochemistry analyses, mice were deeply anesthetized by an intraperitoneal injection of 50 mg kg^−1^ tiletamine/zolazepam (zoletil; Virbac, Italy) and 10 mg kg^−1^ xylazine (Rompum; Bayer, Milan, Italy) and perfused with 4% paraformaldehyde (PFA) in 0.1M phosphate buffered saline (PBS).

Protocols and experimental procedures were carried out according to European Directive 2010/63/EU and approved by the Italian Ministry of Health.

### 2.2. SOD Activity

SOD1 micro-spheres suspended in peanut oil were administered by gavage needle in a single dose of 5U exclusively in the uptake assay for the preliminary evaluation of SOD activity in WT mice. Mice were sacrificed at 0 h, 24 h, 48 h, 72 h. SOD1 activity was also measured in the WT and transgenic animals treated with the SOD-supplemented diet at the end of the treatment. SOD activity was measured in erythrocytes and brain by a colorimetric assay kit (Cayman Chemical Company, Ann Arbor, MI, USA) following the manufacturer’s instructions. Normalization to hemoglobin levels for erythrocytes SOD levels was performed by an automatic hemocytometer (Cell Dyne 400, Abbott, Rome, Italy).

### 2.3. Lipid Peroxidation (LPO) Assay

Lipid peroxidation in erythrocytes and in brain lysates was measured by a commercial colorimetric LPO assay kit (Cayman Chemical Company, Ann Arbor, MI, USA) following the manufacturer’s instructions.

### 2.4. Quantitative Real-Time PCR Analysis

The RNA was extracted with RNeasy Lipid Tissue mini kit (Qiagen, Milan, Italy). 1 mg of total RNA was used for cDNA synthesis and 1 mg of cDNA was used in each real-time reaction, performed in triplicate as previously described [36,37]. Total cDNA amounts were normalized to β-actin (or GAPDH/18S with similar results; not shown), as internal reference, and expressed as fold-increase over control samples. Oligonucleotides used as primers in PCR reactions were previously described [34,36,37,38].

### 2.5. ELISA Protein Analysis

Protein extracts were assayed for Aβ production by Enzyme Linked Immunosorbent Assay (ELISA) using the Aβ 1–40 and Aβ 1–42 Immunoassay kits (BioSource International, Nivelles, Belgium), with good linear sensitivity up to 5 pg/mL (1–40) and 10 pg/mL (1–42).

### 2.6. Immunohistochemistry

Brains were removed from adult mice and post-fixed, overnight. After de-hydration, brain specimens were embedded in Paraplast Tissue Embedding Medium (Leica Biosystems, Milan, Italy) and serially sectioned to obtain sagittal sections (thickness, 9 micron) [46] that were then immunostained as previously described [38]. Briefly, sections were incubated with the anti- Aβ monoclonal antibody DE2B4 (Acris, Herford, Germany), which recognizes Aβ aggregates of senile plaques), at 4 °C overnight. After several washes with PBS, sections were incubated with biotinylated secondary antibody and antibody-antigen complexes were visualized using the Vectastain ABC Elite and DAB Peroxidase Substrate Kits (Vector laboratories, Burlingame, CA, USA), according to manufacturer’s instructions. Adjacent microscopic fields encompassing the cortical and hippocampal regions were acquired using the MetaMorph 5.5 software (Universal Imaging, Downgtown, PA, USA). The abundance of Aβ plaques per field was determined blindly and independently by two investigators and relative plaque area was measured using the MetaMorph 5.5 software tools (Universal Imaging, Downingtown, PA, USA).

### 2.7. Statistical Analysis

**Figure 2:**
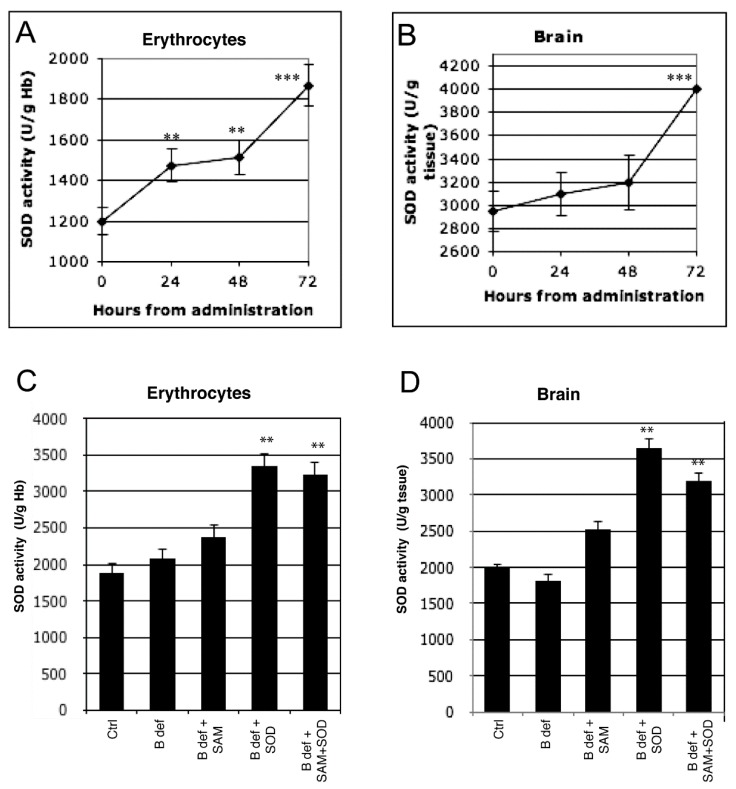
Exogenous SOD supplementation induces endogenous SOD activity. Endogenous SOD activity during 72 h after exogenous administration both in erythrocytes (**A**) and in brain (**B**) of 129 Sv WT mice. Endogenous SOD activity both in erythrocytes (**C**) and in brain (**D**) of TgCRND8 mice fed with SOD-supplemented diet after 2 months of treatment. **: *p* < 0.01; ***: *p* < 0.001.

Two-way Analysis of Variance (ANOVA) followed by Tuckey’s post-test (SPSS software, Milan, Italy) were used for statistical analyses. Dose–response effects were evaluated by linear regression analyses. Histograms indicate the mean value ± standard error of mean (s.e.m). Statistical significance is indicated by asterisks in Figure 2; in the other Figures, due to the complexity of the statistical relationships between the different experimental conditions, tables reporting the statistical differences are presented below the histograms.

## 3. Results

### 3.1. SOD Uptake

To verify that exogenous SOD administration effectively improved endogenous SOD activity, enzymatic tests were performed in erythrocytes and in brain tissue of 129Sv WT mice. SOD activity significantly increased after SOD administration at each time-point (24, 48 and 72 h) compared to control in erythrocytes (Figure 2A). A significant increase of SOD activity in the brain was observed only at 72 h (Figure 2B). SOD activity was also assessed in erythrocytes (Figure 2C) and in brain (Figure 2D) tissue of TgCRND8 mice after 2 months of treatment with SAM and SOD supplemented diet; significant increase of SOD activity was observed in mice treated with SOD supplemented diets.

### 3.2. SAM and SOD Reduce Oxidative Stress

**Figure 3:**
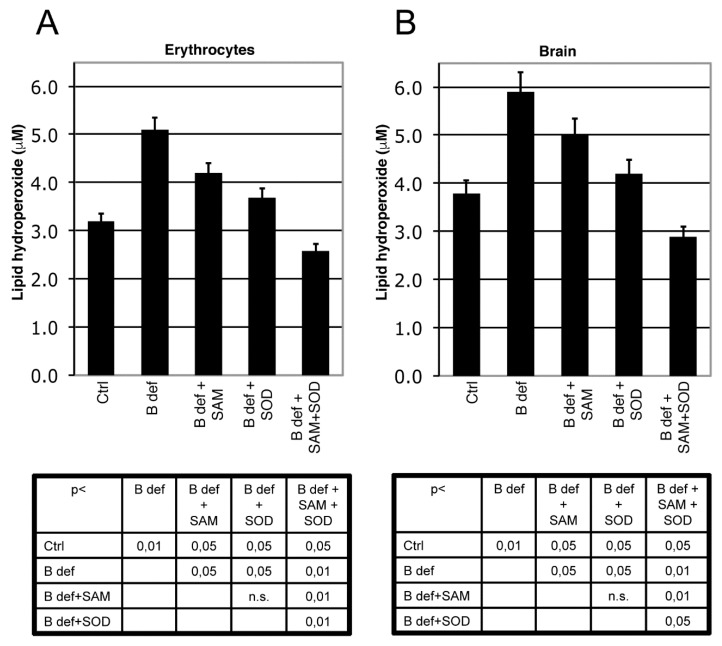
SAM and SOD reduce lipid peroxidation. Lipid peroxidation (LPO) in erythrocytes (**A**) and in brain; (**B**) of TgCRND8 mice treated with control (Ctrl) diet or B vitamin deficient (B def) diet supplemented with SAM and/or SOD. SAM and SOD were orally supplemented at doses of respectively 400 µg/day and 5 U/day when supplemented alone; half doses (200 µg/day and 2.5 U/day) were used for combined supplementation. Statistical significance is listed in the tables below the histograms.

We measured LPO as a marker of oxidative stress in brain tissue and erythrocytes of TgCRND8 mice (Figure 3) and WT littermates (not shown). As previously shown [39], B vitamin deficiency significantly induced oxidative stress, both in brain and erythrocytes (*p* < 0.01 B deficient vs. ctrl), whereas both SAM and SOD supplementation significantly reduced LPO (*p* < 0.05 vs. B deficient). When SAM and SOD were supplemented in combination, LPO was significantly decreased, even besides Ctrl levels, respect to B deficient, B deficient + SAM, B deficient + SOD (erytrocytes: *p* < 0.05 vs. ctrl, *p* < 0.01 vs. B deficient, B deficient + SAM, B deficient + SOD, brain: *p* < 0.05 vs. ctrl, B deficient + SOD, *p* < 0.01 vs. B deficient, B deficient + SAM). LPO levels were also measured in WT mice (not shown) showing results similar to TgCRND8 mice.

### 3.3. SAM and SOD Reduce PSEN1 and BACE Expression

**Figure 4:**
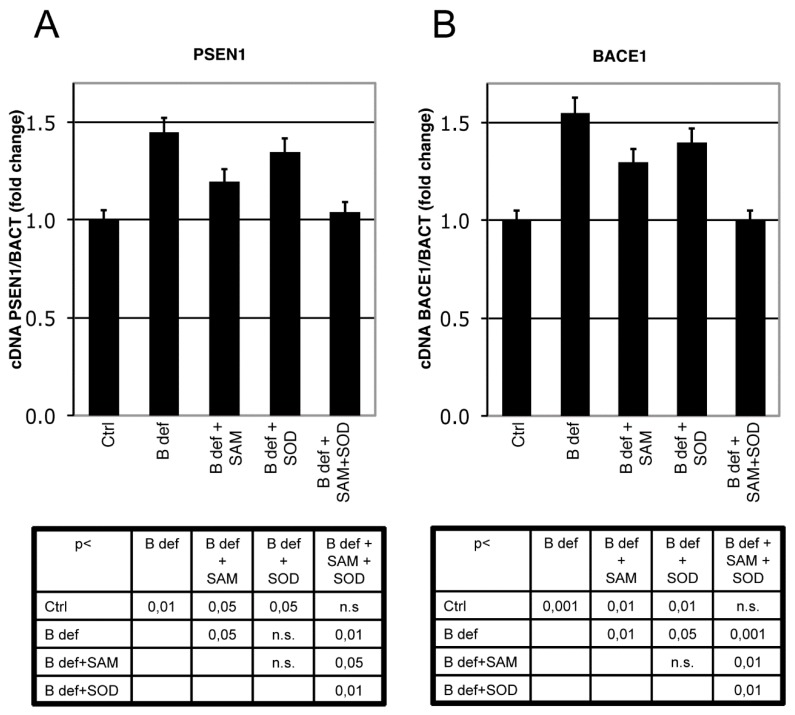
SAM and SOD inhibit PSEN1 and BACE1 expression. Real-Time PCR analysis of PSEN1 (**A**) and BACE1 (**B**) mRNA in TgCRND8 mice brain. Units are expressed as “fold increase” in respect to β-actin, with control indicated as 1 ± s.e.m. Statistical significance is listed in the tables below the histograms. Abbreviation as in Figure 3.

PSEN 1 and BACE1 expression were analyzed by Real-Time PCR analysis in brain of TgCRND8 mice. PSEN1 expression significantly increased in TgCRND8 fed with B vitamin deficient diet, as previously shown [38]. SAM, unlike SOD supplementation, significantly downregulated PSEN1 expression; when SAM and SOD were provided in combination PSEN1 downregulation was even more evident approximating control levels (Figure 4A). BACE1 expression displayed similar regulation (Figure 4B). Indeed, the B vitamin deficient diet significantly increased BACE1 expression, whereas SAM supplementation significantly reduced BACE1 expression, as did SOD supplementation. When SAM and SOD were supplemented in combination, re-established BACE1 expression levels were similar to those of control mice. The diet supplementation with SAM and SOD, provided either alone or in combination, had a similar effect on WT littermates (data not shown).

### 3.4. SAM and SOD Reduce Amyloid Production and Plaque Burden

**Figure 5:**
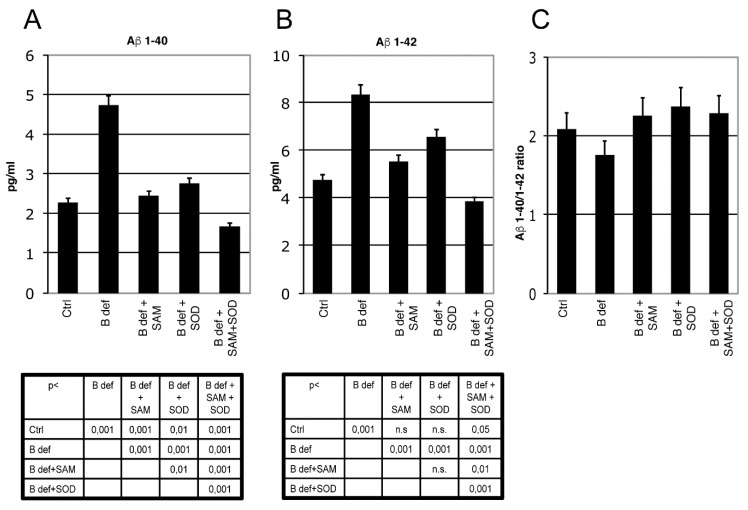
β-Amyloid processing in mice brain. Aβ 1–40 (**A**) and Aβ 1–42 (**B**) ELISA tests performed on brain lysates of TgCRND8 mice; 129Sv mice were used as a negative controls (not shown). Aβ 1–40/1–42 ratio is shown in (**C**). Statistical significance is listed in the tables below the histograms. Abbreviation as in Figure 3.

**Figure 6:**
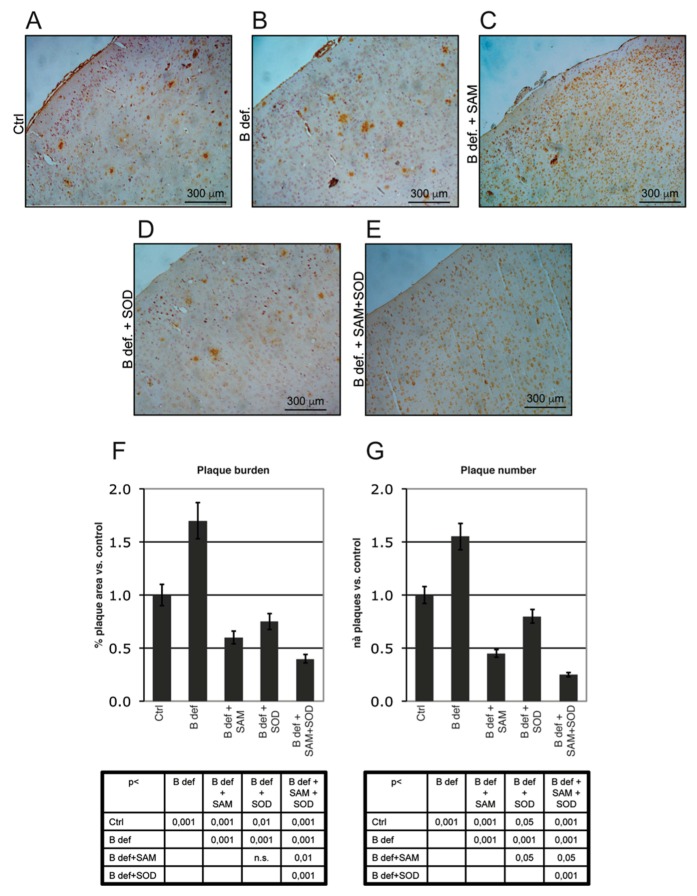
Amyloid plaque burden in TgCRND8 mice brain. Representative micrographs (**A**–**E**) showing amyloid plaques detection in histological sections of the adult mouse brain (Prefrontal Cortex). Magnification 10X. Please note that TgCRND8 mice typically form amyloid plaques at 3 months of age (Ctrl). Plaque area, calculated as % of plaque area/total brain area, (**F**) and plaque number (**G**) values of treated mice are expressed taking that of control mice as 1. Statistical significance is listed in the tables below the histograms. Abbreviations as in Figure 3.

The effect of SAM and SOD, provided either alone or in combination, on Aβ production and aggregation was determined by ELISA and immunohistochemistry assays of brains obtained from TgCRND8 mice belonging to the various experimental groups (Figure 5 and Figure 6). ELISA assays confirmed our previous finding demonstrating that Aβ 1–40 (Figure 5A) and Aβ 1–42 (Figure 5B) production was significantly increased in the brain of mice fed with a vitamin B-deficient diet, and that SAM supplementation counteracted this effect [38]. Although these findings were already shown in our previous study, we here repeated the assays for a direct comparison between SAM and SOD supplementation. Figure 5A,B shows that SOD administration significantly contrasted Aβ peptides overproduction and that the supplementation of SAM and SOD in combination displayed a very strong efficacy, since it reduced Aβ 1–40 and Aβ 1–42 below control levels.

The ratio of Aβ 1–40/1–42 (Figure 5C) was not influenced by the different experimental conditions, since the two amyloid peptides were apparently modulated similarly.

Since endogenous murine amyloid is expressed at very low levels and the ELISA kit we used is specific for the peptide of human origin, extracts from brain of 129Sv mice were used as negative controls (not shown).

Plaque burden was also evaluated by immunohistochemistry. Figure 6A–E shows representative stained sections of the prefrontal cortex obtained from TgCRND8 mice treated for 2 months with control or B vitamin-deficient diet, with or without SAM and SOD, provided either alone or in combination. Plaque burden (area and number), determined by Metamorph Software-assisted tools, is reported in Figure 6F,G respectively. Results are expressed as fold changes vs. the control diet condition, assumed = 1; plaque area was calculated as percent of brain area occupied by plaques respect to the total area analyzed. B vitamin-deficiency considerably increased amyloid deposition whereas SAM reduced plaques to control-like levels, as previously reported [38]. SOD supplemented to B vitamin deficient diet determined a reduction of plaque area similar to that elicited by diet supplementation with SAM, having a less intense effect on plaque number (Figure 6G, *p* < 0.05). Interestingly, the combined supplementation of SAM and SOD was associated with the greatest reduction of both plaques area and number, confirming the synergistic effect as already indicated by the ELISA assays.

## 4. Discussion

Although the etiology of LOAD is multifactorial and poorly understood, the combination of genetic and environmental factors, including nutrition, plays a central role in the onset and progression of the disease. By consequence, nutritional intervention can represent a relevant route to achieve beneficial effects in AD treatment, at least in combination with drug therapy. In the present paper, we provide evidence that two “nutraceutical” molecules, SAM and SOD, retain the ability to synergistically contrast a large spectrum of AD-related mechanisms with virtually no side-effects.

SAM, either as drug or nutritional supplement, is widely used for the treatment of liver diseases, depression and ostheoarthtitis, with high tolerability and the very few side effects [47] limited to mild gastrointestinal upsets, usually resolved after lowering doses. Oral formulations, in the form of coated gastroresistant tablets and capsules, allow SAM irritating activity on the gastric mucosa to be overcome, also hiding its unpleasant taste. SOD is widely used as nutritional supplements to contrast oxidative stress and ROS, with no side effects reported [48]. Nutritional lifestyle regulates the intake and bioavailability of metabolites and cofactors. B vitamins and methyl-rich nutrients are particularly important since they are involved in the “one-carbon” metabolism, one of the access points for the environment to modulate physiological aging processes toward neurodegeneration [49,50,51]. One-carbon metabolism is a complex metabolic pathway involving three interrelated biochemical routes: the folate cycle, the transsulfuration pathway and the methionine cycle (Figure 1). Briefly, Hcy can be converted to Cystathionine and then to GSH by transsulfuration or remethylated to methionine by vitamin B12-dependent enzyme Methionine Synthase (MS), which uses 5-methyltetrahydrofolate as co-substrate [30,31]. Methionine is then transformed to SAM which, after donating a methyl group to various molecules (like DNA, RNA, proteins) is converted to SAH, further hydrolyzed to Hcy and Adenosine in a reversible reaction. The alteration of one-carbon metabolism can occur at either genetic levels or following alterations in B vitamins levels. Aberrations in one carbon metabolism, high Hcy and low B vitamin levels are positively associated with LOAD [12,33,52,53]. For instance, the impairment of either remethylation or transsulfuration pathways can lead to hyperhomocysteinemia, alteration of GSH metabolism and increase of SAH levels, a potent inhibitor of SAM dependent methyl trasnsferases. This imbalance affects DNA methylation, the main epigenetic control of gene expression, along with redox homeostasis, exacerbating AD-like features as we previously demonstrated in the TgCRND8 AD mice model [34,35,36,37,39]. The B vitamin nutritional deficiency applied to the AD transgenic animal model used in our studies was designed to specifically induce one-carbon metabolism alterations resulting in DNA methyltransferase impairment and DNA demethylase improvement, PSEN1 hypomethylation, PSEN1 and BACE1 upregulation and plaque deposition [34,36,37]. On the other hand, we have shown that SAM supplementation is able to contrast the observed exacerbation of all AD-like features induced by B vitamin deficiency, also counteracting the manifestation of cognitive deficit [38].

The alteration of GSH metabolism caused by the impairment of the transsulfuration pathway suggests that, in addition to hypomethylation, altered redox homeostasis is a mechanism through which one-carbon metabolism is involved in brain diseases, and that an interaction exists between oxidative stress and DNA methylation [32,54,55]. Moreover, it is well known that methyl deficient diets and hyperhomocysteinemia (HHcy) induce oxidative stress that, in a sort of vicious cycle, may inhibit DNA methylation [56]. It is reported that folate deficiency is associated with HHcy and decreased activity of Cu-Zn SOD and glutathione peroxidase [33,57]. Oxidative stress has been suggested as a key process involved in AD pathogenesis and in the exacerbation of AD features, such as Aβ plaques and tangles, by oxidizing several key proteins and modulating their activity [2,16,58,59]. Moreover, increased levels of markers of oxidative damage have been shown in the blood, cerebrospinal fluid (CSF) and brain of AD patients [3,17,18]. The oxidation of proteins involved in the production and/or regulation of amyloid can directly affect Aβ production and oligomerization. Finally, several studies evidenced that Aβ aggregates can generate free radicals and an oxidative microenvironment, leading to oxidative stress and neurotoxicity [22,23]. Taken together, these findings suggest a bidirectional inter-relation between Aβ and oxidative stress that may result in a positive feedback pathway. Several studies have underlined that the loss of the antioxidant enzyme Cu-Zn-superoxide dismutase in mice causes several accelerated age-related pathologies and lifespan reduction [60,61,62,63]. Even if the contribution of SOD to AD pathogenesis appears controversial [28], some studies suggest that MnSOD plays a protective role during AD development, and MnSOD deficiency increases Aβ levels and accelerates the onset of behavioral alteration in APP transgenic mice [64,65].

In a previous study we demonstrated that SAM prevents oxidative stress by modulating GSH levels and SOD activity in TgCRND8 mice fed with a vitamin B-deficient diet [39]. The SOD ability to counteract oxidative injuries and the finding that one-carbon metabolism represents the link between DNA methylation and oxidative stress [32,66], prompted us to study the effect of the combined supplementation of SAM and SOD exploiting our model of AD features induced by B vitamin deficiency. Since the cellular response to oxidative stress typically involves alteration in thiols content, we recently evaluated the effect of SAM and SOD supplementation on plasma thiol levels in the same animal model, finding a significant decrease of thiol levels when the B vitamin deficient diet was supplemented with SAM + SOD and SOD alone, the latter showing the greatest effect [40].

In order to ensure SOD bioavailability by preventing possible degradation due to its high chemical-physical instability, the dried SOD powder was coated by a gastroresistant formulation. Like the majority of proteins, SOD is not able to resist the gastric environment and its dismutase activity is not preserved during its transit through the stomach unless it is gastro protected. In this study the gastro protection was achieved by a fluid bed coating of SOD granules with a gastro resistant film. We verified that exogenous SOD orally supplemented was able to increase endogenous SOD activity in WT mice, both in blood and in brain. B vitamin deficiency-dependent HHcy in mice [40] caused an impaired DNA methylation together with a marked oxidative stress, as evidenced by high LPO levels measured in erythrocytes and in brain of mice. SAM and SOD were able to significantly reduce LPO levels although only the combined SAM and SOD supplementation decreasing LPO to control levels, showing an unexpected synergistic effect in counteracting oxidative imbalance. We already demonstrated that SAM potentiated SOD and Glutathione S-transferase activities [39], providing additional antioxidant power to the combination of SAM and SOD. Furthermore SAM is able to modulate the competing remethylation and transsulfuration pathways: low SAM levels are able to provide methionine and SAM synthesis by shifting Hcy metabolism towards remethylation, while high SAM levels strengthen the transsulfuration pathway, and then GSH production, also being SAM and SAH allosteric activators of Cystathionine-β-synthase (CBS) [31]. Findings of this study also show a synergistic effect of the SAM and SOD combination in inhibiting PSEN1 and BACE1 expression. Interestingly, while SAM inhibited both PSEN1 and BACE1 as previously demonstrated [36,37,38], SOD alone was only able to counteract BACE1 overexpression. This differential effect is in agreement with the observation that, unlike PSEN1, BACE1 expression is regulated by oxidative stress but not by DNA methylation [67]. Therefore, SAM and SOD combination seems to synergistically affect both oxidation and DNA methylation leading to the most efficacious inhibition of both genes.

Inhibition of amyloid processing and reduction of plaque spreading have been evaluated as downstream markers of Alzheimer’s-like features induced by B vitamin deficiency. Again, the combined supplementation of SAM and SOD showed synergistic effect on reducing Aβ production and amyloid plaques burden in TgCRND8 mice. The unexpected interaction and super additive effect of the SAM and SOD combination was quantitatively assessed by the method of isoboles, which is the most common method for the quantitative assessment of unusual interaction between agonist drugs [68]. This analysis was performed on plaque burden data, since this is the main parameter used to quantify senile plaques spreading (data not shown) and highlighted that the combined effect was greater than that predicted for the individual potencies of the two molecules, despite the use of lower doses. Drug combination therapy is quite common in the treatment of many diseases and may represent a promise even for LOAD; furthermore, the use of low doses of the compounds may reduce possible adverse reactions. A recent paper, indeed, showed the potential of low doses of SAM in AD mice respect to high doses [69].

Up to now, no FDA-approved antioxidant therapy exists. This is mainly a consequence of the unconfirmed promising pre-clinical results in the following clinical trials [2,4]. An attempt to improve the outcome of AD clinical trials is represented by the application of therapies combining multiple compounds, targeting different pathways altered in AD. In one of these trials, a nutraceutical formulation consisting of six vitamins and nutraceuticals (folic acid, vitamin B12, vitamin E, Acetyl-L-Carnitine, NAC, and SAM) ameliorated the cognitive performance in community-dwelling adults without dementia [70], underscoring the importance of nutritional supplements in the elderly. A similar formulation was used in a one-year open label pilot study in early-stage AD, showing effectiveness in preserving cognitive abilities if started at early stage AD [71]. Moreover, patients with moderate AD concurrently treated with donepezil and a combination of the most common antioxidant (carnosine, coenzyme Q10, vitamin E, vitamin C, beta-carotene, selenium, L-cysteine, Ginkgo Biloba and vitamins B) displayed a significant improvement in MMSE II score respect to a group treated with donepezil and placebo. It is worth noting a multivitamin (vitamins B6 and B12 and folic acid) supplementation approach to mild-to-moderate AD patients already taking AchE inhibitors did not provide a specific improvement [4].

The novelty of the treatment we assessed in this study consists of the combination of two powerful antioxidant and methylation agents, such as SOD and SAM, respectively, which likely contrast the AD-like features in TgCRND8 acting both on oxidative balance and methylation.

We show that the efficacy of SAM and SOD at half doses displayed unexpected synergistic effects, improving the antioxidant power, reducing oxidative stress, inhibiting PSEN1 and BACE1 overexpression and significantly reducing amyloid production and plaque burden. In this regard, it is worth noting that SAM + SOD treatment reduces AD-like features in B vitamin deficient mice even below control levels (i.e., in TgCRND8 mice treated with the control diet). This observation led us to infer that the treatment could potentially have efficacy in patients also in the absence of B vitamin deficiency and/or HHcy and even in familial forms of the disease. This idea is reinforced by the observation that non-AD related molecular response (LPO, gene expression) is similarly modulated in WT (not shown) and TgCRND8 mice. SAM and SOD are used either as a drug or as a nutritional supplement in humans in several countries with low side effects that could be further minimized by the use of low doses. The observed effect of exogenous SOD in the brain is surprising since, although gastro-protected, large proteins like SOD are usually degraded during intestinal absorption. The understanding of exogenous SOD efficacy and mechanisms of action requires further investigation, but it is noteworthy that we could demonstrate that oral supplementation is associated with increased SOD activity in tissues.

On the other hand, we already demonstrated that exogenous SAM [38] and SOD administrations were accompanied by an increase of their levels and/or activities in the brain. This is a very relevant finding because many other compounds, often displaying promising effects in in vitro models, did not show a similar efficacy in animal models due to their poor cerebral uptake.

Results of this study further confirm the central role of one carbon metabolism as a key pathway mediating methylation and oxidative imbalance in LOAD, emphasizing the need for additional studies aimed at evaluating the use of the synergistic combination of SAM and SOD as potential therapeutic/coadjuvant agents in AD treatment.

## Supplementary Materials

The following are available online at http://www.mdpi.com/2076-3921/6/4/76/s1, Table S1: Quali-quantitative composition of SOD microspheres.

## References

[1] Prince M., Bryce R., Albanese E., Wimo A., Ribeiro W., Ferri C.P. (2013). The global prevalence of dementia: A systematic review and metaanalysis. Alzheimers Dement..

[2] Tönnies E., Trushina E. (2017). Oxidative Stress, Synaptic Dysfunction, and Alzheimer’s Disease. J. Alzheimers Dis..

[3] García-Blanco A., Baquero M., Vento M., Gil E., Bataller L., Cháfer-Pericás C. (2017). Potential oxidative stress biomarkers of mild cognitive impairment due to Alzheimer disease. J. Neurol. Sci..

[4] Giulietti A., Vignini A., Nanetti L., Mazzanti L., Di Primio R., Salvolini E. (2016). Alzheimer’s Disease Risk and Progression: The Role of Nutritional Supplements and their Effect on Drug Therapy Outcome. Curr. Neuropharmacol..

[5] Iqbal K., Grundke-Iqbal I. (2010). Alzheimer’s disease, a multifactorial disorder seeking Multitherapies. Alzheimers Dement..

[6] Ballard C., Gauthier S., Corbett A., Brayne C., Aarsland D., Jones E. (2011). Alzheimer’s disease. Lancet.

[7] Migliore L., Coppede F. (2009). Genetics, environmental factors and the emerging role of epigenetics in neurodegenerative diseases. Mutat. Res..

[8] Hasan M.K., Mooney R.P. (2011). The predisposing factors, biological markers, neuroimaging techniques and medical complications associated with Alzheimer’s disease. W. Va. Med. J..

[9] Herrmann W., Obeid R. (2011). Homocysteine: A biomarker in neurodegenerative diseases. Clin. Chem. Lab. Med..

[10] Nunomura A., Castellani R.J., Zhu X., Moreira P.I., Perry G., Smith M.A. (2006). Involvement of oxidative stress in Alzheimer disease. J. Neuropathol. Exp. Neurol..

[11] Meraz-Ríos M.A., Franco-Bocanegra D., Toral Rios D., Campos-Peña V. (2014). Early Onset Alzheimer’s Disease and Oxidative Stress. Oxid. Med. Cell. Longev..

[12] Smith A.D., Refsum H. (2016). Homocysteine, B Vitamins, and Cognitive Impairment. Annu. Rev. Nutr..

[13] Manoharan S., Guillemin G.J., Abiramasundari R.S., Essa M.M., Akbar M., Akbar M.D. (2016). The Role of Reactive Oxygen Species in the Pathogenesis of Alzheimer’s Disease, Parkinson’s Disease, and Huntington’s Disease: A Mini Review. Oxid. Med. Cell. Longev..

[14] Huang W.J., Zhang X., Chen W.W. (2016). Role of oxidative stress in Alzheimer’s disease. Biomed. Rep..

[15] Bonda D.J., Wang X., Lee H.G., Smith M.A., Perry G., Zhu X. (2014). Neuronal failure in Alzheimer’s disease: A view through the oxidative stress looking-glass. Neurosci. Bull..

[16] Lovell M.A., Markesbery W.R. (2007). Oxidative damage in mild cognitive impairment and early Alzheimer’s disease. J. Neurosci. Res..

[17] Casado A., Lopez-Fernandez M.E., Casado M.C., De La Torre R. (2008). Lipid peroxidation and antioxidant enzyme activities in vascular and Alzheimer dementias. Neurochem. Res..

[18] Chang Y.T., Chang W.N., Tsai N.W., Huang C.C., Kung C.T., Su Y.J., Lin W.C., Cheng B.C., Su C.M., Chiang Y.F. (2014). The Roles of Biomarkers of Oxidative Stress and Antioxidant in Alzheimer’s Disease: A Systematic Review. BioMed Res. Int..

[19] Bradley-Whitman M.A., Lovell M.A. (2015). Biomarkers of lipid peroxidation in Alzheimer disease (AD): An update. Arch. Toxicol..

[20] Coppedè F., Migliore L. (2015). DNA damage in neurodegenerative diseases. Mutat. Res..

[21] França M.B., Lima K.C., Eleutherio E.C. (2017). Oxidative Stress and Amyloid Toxicity: Insights from Yeast. J. Cell. Biochem..

[22] Butterfield D.A., Swomley A.M., Sultana R. (2013). Amyloid beta-peptide (1–42)-induced oxidative stress in Alzheimer disease: Importance in disease pathogenesis and progression. Antioxid. Redox Signal..

[23] Saharana S., Mandal P.K. (2014). The Emerging Role of Glutathione in Alzheimer’s Disease. J. Alzheimers Dis..

[24] B´elanger M., Allaman I., Magistretti P.J. (2011). Brain energy metabolism: Focus on astrocyte-neuron metabolic cooperation. Cell Metab..

[25] Mariani E., Polidori M.C., Cherubini A., Mecocci P. (2005). Oxidative stress in brain aging, neurodegenerative and vascular disease: An overview. J. Chromatogr. B..

[26] Lovell M.A., Xie C., Markesbery W.R. (1998). Decreased glutathione transferase activity in brain and ventricular fluid in Alzheimer’s disease. Neurology.

[27] Johnson W.M., Wilson-Delfosse A.L., Mieyal J.J. (2012). Dysregulation of Glutathione Homeostasis in Neurodegenerative Diseases. Nutrients.

[28] Murakami K., Murata N., Noda Y., Tahara S., Kaneko T., Kinoshita N., Hatsuta H., Murayama S., Barnham K.J., Irie K. (2011). SOD1 (Copper/Zinc Superoxide Dismutase) deficiency drives Amyloid β protein oligomerization and memory loss in mouse model of Alzheimer disease. J. Biol. Chem..

[29] Massaad C.A., Washington T.M., Pautler R.G., Klann E. (2009). Overexpression of SOD-2 reduces hippocampal superoxide and prevents memory deficits in a mouse model of Alzheimer’s disease. Proc. Natl. Acad. Sci. USA.

[30] Finkelstein J.D. (1998). The metabolism of homocysteine: Pathways and regulation. Eur. J. Pediatr..

[31] Finkelstein J.D. (2007). Metabolic regulatory properties of S-adenosylmethionine and S-adenosylhomocysteine. Clin. Chem. Lab. Med..

[32] Fleming J.L., Phiel C.J., Toland A.E. (2012). The role for oxidative stress in aberrant DNA methylation in Alzheimer’s disease. Curr. Alzheimer Res..

[33] Troesch B., Weber P., Mohajeri M.H. (2016). Potential Links between Impaired One-Carbon Metabolism Due to Polymorphisms, Inadequate B-Vitamin Status, and the Development of Alzheimer’s Disease. Nutrients.

[34] Fuso A., Nicolia V., Cavallaro R.A., Ricceri L., D’Anselmi F., Coluccia P., Calamandrei G., Scarpa S. (2008). B-vitamin deprivation induces hyperhomocysteinemia and brain S-adenosylhomocysteine, depletes brain S-adenosylmethionine, and enhances PS1 and BACE expression and amyloid-beta deposition in mice. Mol. Cell. Neurosci..

[35] Nicolia V., Fuso A., Cavallaro R.A., Di Luzio A., Scarpa S. (2010). B vitamin deficiency promotes tau phosphorylation through regulation ofGSK3b and PP2A. J. Alzheimers Dis..

[36] Fuso A., Nicolia V., Pasqualato A., Fiorenza M.T., Cavallaro R.A., Scarpa S. (2011). Changes in Presenilin 1 gene methylation pattern in diet-induced B vitamindeficiency. Neurobiol. Aging.

[37] Fuso A., Nicolia V., Cavallaro R.A., Scarpa S. (2011). DNA methylase and demethylaseactivities are modulated by one-carbon metabolism in Alzheimer’s disease models. J. Nutr. Biochem..

[38] Fuso A., Nicolia V., Ricceri L., Cavallaro R.A., Isopi E., Mangia F., Fiorenza M.T., Scarpa S. (2012). S-adenosylmethionine reduces the progress of the Alzheimer-like features induced by B-vitamin deficiency in mice. Neurobiol. Aging.

[39] Cavallaro R.A., Fuso A., Nicolia V., Scarpa S. (2010). S-adenosylmethionine prevents oxidative stress and modulates glutathione metabolism in TgCRND8 mice fed a B-vitamin deficient diet. J. Alzheimers Dis..

[40] Persichilli S., Gervasoni J., Di Napoli A., Fuso A., Nicolia V., Giardina B., Scarpa S., Desiderio C., Cavallaro R.A. (2015). Plasma thiols levels in Alzheimer’s disease mice under diet-induced hyperhomocysteinemia: Effect of S-adenosylmethionine and superoxide-dismutase supplementation. J. Alzheimers Dis..

[41] Olajide O.J., Yawson E.O., Gbadamosi I.T., Arogundade T.T., Lambe E., Obasi K., Lawal I.T., Ibrahim A., Ogunrinola K.Y. (2017). Ascorbic acid ameliorates behavioural deficits and neuropathological alterations in rat model of Alzheimer’s disease. Environ. Toxicol. Pharmacol..

[42] Persson T., Popescu B.O., Cedazo-Minguez A. (2014). Oxidative Stress in Alzheimer’s Disease: Why Did AntioxidantTherapy Fail?. Oxid. Med. Cell. Longev..

[43] Conte V., Uryu K., Fujimoto S., Yao Y., Rokach J., Longhi L., Trojanowski J.Q., Lee V.M.-Y., McIntosh T.K., Praticò D. (2004). Vitamin E Reduces Amyloidosis and Improves Cognitive Function in Tg2576 Mice Following Repetitive Concussive Brain Injury. J. Neurochem..

[44] Lloret A., Badía M.-C., Mora N.J., Pallardó F.V., Alonso M.-D., Viña J. (2009). Vitamin E Paradox in Alzheimer’s Disease: It Does Not Prevent Loss of Cognition and May Even Be Detrimental. J. Alzheimers Dis..

[45] Li F.J., Shen L., Ji H.F. (2012). Dietary Intakes of Vitamin E, Vitamin C, and Β-Carotene and Risk of Alzheimer’s Disease: A Meta- Analysis. J. Alzheimers Dis..

[46] Canterini S., Bosco A., Carletti V., Fuso A., Curci A., Mangia F., Fiorenza M.T. (2012). Subcellular TSC22D4 localization in cerebellum granule neurons of the mouse depends on development and differentiation. Cerebellum.

[47] Sharma A., Gerbarg P., Bottiglieri T., Massoumi L., Carpenter L.L., Lavretsky H., Muskin P.R., Brown R.P., Mischoulon D., As Work Group of the American Psychiatric Association Council on Research (2017). S-Adenosylmethionine (SAMe) for Neuropsychiatric Disorders: A Clinician-Oriented Review of Research. J. Clin. Psychiatry.

[48] Romao S. (2015). Therapeutic value of oral supplementation with melon superoxide dismutase and wheat gliadin combination. Nutrition.

[49] Lahiri D.K., Maloney B., Basha M.R., Ge Y.W., Zawia N.H. (2007). How and when environmental agents and dietary factors affect the course of Alzheimer’s disease: The “LEARn” model (latent early-life associatedregulation) may explain the triggering of AD. Curr. Alzheimer Res..

[50] Wu J., Basha M.R., Zawia N.H. (2008). The environment, epigenetics and amyloidogenesis. J. Mol. Neurosci..

[51] Coppedè F., Tannorella P., Pezzini I., Migheli F., Ricci G., Caldarazzo lenco E., Piaceri I., Polini A., Nacmias B., Monzani F., Sorbi S., Siciliano G., Migliore L. (2012). Folate, homocysteine, vitamin B12, and polymorphisms of genes participating in one-carbon metabolism in late-onset Alzheimer’s disease patients and healthy controls. Antioxid. Redox Signal..

[52] Luchsinger J.A., Tang M.X., Miller J., Green R., Mehta P.D., Mayeux R. (2007). Relation of plasma homocysteine to plasma amyloid beta levels. Neurochem. Res..

[53] Haan M.N., Miller J.W., Aiello A.E., Whitmer R.A., Jagust W.J., Mungas D.M., Allen L.H., Green R. (2007). Homocysteine, B vitamins, and the incidence of dementia and cognitive impairment: Results from the Sacramento Area Latino Study on Aging. Am. J. Clin. Nutr..

[54] Pocernich C.B., Butterfield D.A. (2011). Elevation of glutathione as a therapeutic strategy in Alzheimer disease. Biochim. Biophys. Acta.

[55] Zhang C., Rodriguez C., Spaulding J., Aw T.Y., Feng J. (2012). Age-dependent and tissue-related glutathione redox status in a mouse model of Alzheimer’s disease. J. Alzheimers Dis..

[56] Takumi S., Okamura K., Yanagisawa H., Sano T., Kobayashi Y., Nohara K. (2015). The effect of a methyl-deficient diet on the global DNA methylation and the DNA methylation regulatory pathways. J. Appl. Toxicol..

[57] Huang R.-F.S., Hsu Y.-C., Lin H.-L., Yang F.L. (2001). Folate depletion and elevated plasma homocysteine promo teoxidative stress in rat livers. J. Nutr..

[58] Aksenov M.Y., Aksenova M.V., Butterfield D.A., Geddes J.W., Markesbery W.R. (2001). Protein oxidation in the brain in Alzheimer’s disease. Neuroscience.

[59] Keller J.N., Schmitt F.A., Scheff S.W., Ding Q., Chen Q., Butterfield D.A., Markesbery W.R. (2005). Evidence of increased oxidative damage in subjects with mild cognitive impairment. Neurology.

[60] Reddy V.N., Kasahara E., Hiraoka M., Lin L.R., Ho Y.S. (2004). Effects of variation in superoxide dismutases (SOD) on oxidative stress and apoptosis in lens epithelium. Exp. Eye Res..

[61] Elchuri S., Oberley T.D., Qi W., Eisenstein R.S., Jackson Roberts L., Van Remmen H., Epstein C.J., Huang T.T. (2005). Cu ZnSOD deficiency leads to persistent and widespread oxidative damage and hepatocarcinogenesis later in life. Oncogene.

[62] Muller F., Song W., Liu Y., Chaudhuri A., Pieke-Dahl S., Strong R., Huang T.T., Epstein C.J., Roberts L.J., Csete M. (2006). Absence of CuZn superoxide dismutase leads to elevated oxidative stress and acceleration of age-dependent skeletal muscle atrophy. Free Radic. Biol. Med..

[63] Fischer L.R., Glass J.D. (2010). Oxidative stress induced by loss of Cu, Zn-superoxide dismutase (SOD1) or superoxide-generating herbicides causes axonal degeneration in mouse DRG cultures. Acta Neuropath..

[64] Esposito L., Raber J., Kekonius L., Yan F., Yu G.Q., Bien-Ly N., Puoliväli J., Scearce-Levie K., Masliah E., Mucke L. (2006). Reduction in mitochondrial superoxide dismutase modulates Alzheimer’s disease-like pathology and accelerates the onset of behavioral changes in human amyloid precursor protein transgenic mice. J. Neurosci..

[65] Feng Y., Wang X. (2012). Antioxidant therapies for Alzheimer’s disease. Oxid. Med. Cell. Longev..

[66] Niedzwiecki M.M., Hall M.N., Liu X., Oka J., Harper K.N., Slavkovich V., Ilievski V., Levy D., van Geen A., Mey J.L. (2013). Blood glutathione redox status and global methylation of peripheral blood mononuclear cell DNA in Bangladeshi adults. Epigenetics.

[67] Tong Y., Zhou W., Fung V., Christensen M.A., Qing H., Sun X., Song W. (2005). Oxidative stress potentiates BACE1 gene expression and Aβ generation. J. Neural Transm..

[68] Tallarida R.J. (2011). Quantitative methods for assessing drug synergism. Genes Cancer.

[69] Do Carmo S., Hanzel C.E., Jacobs M.L., Machnes Z., Iulita M.F., Yang J., Yu L., Ducatenzeiler A., Danik M., Breuillaud L.S. (2016). Rescue of Early bace-1 and Global DNA Demethylation by S-Adenosylmethionine Reduces Amyloid Pathology and Improves Cognition in an Alzheimer’s Model. Sci. Rep..

[70] Chan A., Remington R., Kotyla E., Lepore A., Zemianek J., Shea T.B. (2010). A vitamin/nutriceutical formulation improves memory and cognitive performance in community-dwelling adults without dementia. J. Nutr. Health Aging.

[71] Chan A., Paskavitz J., Remington R., Rasmussen S., Shea T.B. (2008). Efficacy of a vitamin/nutriceutical formulation for early stage Alzheimer Disease: A 1 year open label pilot study with an 16 months care-giver extension. Am. J. Alzheimers Dis. Other Dement..

