# Supplementary material for: S-Adenosylmethionine and Superoxide Dismutase 1 Synergistically Counteract Alzheimer’s Disease Features Progression in TgCRND8 Mice
Source: Antioxidants (Basel). 2017 Sep 30;6(4):76. doi: 10.3390/antiox6040076 (PMC5745486; doi:10.3390/antiox6040076)
Supplement: Supplementary file 1 [file antioxidants-06-00076-s001.pdf]

antioxidants

Table S1. Quali-quantitative composition of SOD microspheres.

Components Description	Weight (g)	Function
**Active Ingredient(s)**		
Freeze dried SOD powder	260.00	Active Ingredient
**Excipients**		
Stearic acid p.f. 54–56 °C	100.00	Diluent agent
Calcium cloride	10.00	Dissecant agent
**melted 15 min at 60 °C**		
Mannitol	100.00	Diluent
Microcrystalline cellulose pH 112	30.00	Binder
**pre-compressed (I)**		
Stearic acid p.f. 54–56 °C	20.00	Diluent agent
**pre-compressed (II)**		
**Weight**	**520.00**	
**Excipients (coating)**		
Arginine Shellac (SSB 57 Luna)	400.00	Coating agent (gastro-resistant)
Triethylcitrate	30.00	Platicizer
Talc	70.00	Lubrificant
Titanium dioxide	20.00	Opacizant
**Total weight**	**1040.00**	
